# Property Enhancement of Recycled Coarse Aggregate and Its Concrete under CO_2_-Accelerated Curing Treatment

**DOI:** 10.3390/ma17174194

**Published:** 2024-08-24

**Authors:** Yingying Li, Jia Long, Xiang Chen

**Affiliations:** National Engineering Research Center for Inland Waterway Regulation, School of River and Ocean Engineering, Chongqing Jiaotong University, 66 Xuefu Road, Nan’an District, Chongqing 400074, China; 19923545177@163.com

**Keywords:** recycled coarse aggregate, recycled coarse aggregate concrete, CO_2_-accelerated carbonation, initial moisture condition (IMC), aggregate particle size (APS), modification effect

## Abstract

The poor properties of recycled coarse aggregate (RCA) and recycled coarse aggregate concrete (RCAC) are considered key constraints hindering the reuse of this waste resource in marine engineering. The CO_2_-based accelerated carbonation method, which utilizes the alkali aggregate properties of RCA to achieve CO_2_ uptake and sequestration while significantly enhancing its properties, has attracted widespread attention. However, the degree of improvement in the properties of RCA under different initial moisture conditions (IMCs) and aggregate particle sizes (APSs) after CO_2_-accelerated carbonation remains unclear. Moreover, the quantitative effect of carbonated recycled coarse aggregate (CRCA), which is obtained from RCA samples with the optimal initial moisture conditions, on the improvement of RCAC under optimal accelerated carbonation modification conditions still needs to be studied in depth. For this investigation, a CO_2_-accelerated carbonation experiment was carried out on RCA samples with different IMCs and APSs, and the variations in the properties of RCA with respect to its IMC and APS were assessed. The degree of accelerated carbonation modification of RCA under different IMCs and APSs was quantified, and the optimal initial moisture conditions for enhancing the properties of the RCA were confirmed. By preparing concrete specimens based on the natural coarse aggregate, RCA, and CRCA with the best initial moisture conditions (considering the same concrete–water proportion), the effect of CRCA on the workability, mechanical properties, and durability of the corresponding concrete specimen was determined. The findings of this study can be used to effectively promote the sustainable development of marine science and engineering in the future and contribute to global dual-carbon goals, which are of great practical significance and scientific value.

## 1. Introduction

With the development of the international marine economy and the rapid rise of the marine industry, the construction and restoration of numerous structures along coastlines have greatly increased the demand for concrete raw materials. At the same time, this has also led to a yearly increase in the amount of discarded concrete generated from marine engineering, seriously affecting the marine ecological environment [1]. In addition, large amounts of CO_2_ are produced during the production and preparation of concrete. Taking China as an example, with the development of China’s marine industry, marine carbon emissions are still increasing annually. From 2008 to 2019, China’s total marine carbon emissions have been estimated to have reached approximately 24.55 million tons [2]. How to reduce marine carbon emissions and convert marine waste into reusable resources has now become a major problem in the field of marine science that urgently needs to be researched and solved.

The production and preparation of recycled coarse aggregate (RCA, see in Table A1 of Appendix A) based on the reuse of waste concrete and its use to partially or completely replace natural coarse aggregate (NCA, see in Table A1 of Appendix A) in concrete is an effective way to realize the sustainable use of marine debris [3,4,5,6]. The research and development of marine waste concrete reuse technology can not only greatly reduce the exploitation of natural resources but can also effectively promote the sustainable development of marine science in the future and facilitate the early realization of global carbon peak and carbon neutrality goals, which are of great scientific value and practical significance. However, due to the loose pore structure of old mortar and the old interfacial transition zone (OITZ, see in Table A1 of Appendix A) within RCA, it has poorer properties than NCA in terms of apparent density, water absorption, and other properties [7,8,9,10]. Therefore, it is difficult to satisfy the actual engineering requirements when using RCA without any treatment to replace NCA. Furthermore, the workability, mechanical properties, and durability of recycled coarse aggregate concrete (RCAC, see in Table A1 of Appendix A) prepared using RCA without any modification are worse than those of natural coarse aggregate concrete (NCAC, see in Table A1 of Appendix A). This fatal flaw seriously limits the future popularization and application of RCA in marine engineering fields.

Some scholars have conducted work related to the life cycle assessment of waste concrete powder, optimized the carbonation process, and optimized the recycling of waste concrete powder through the use of carbon footprint and life cycle assessment [11]. To effectively improve the workability, mechanical properties, and durability of RCAC prepared from RCA, reduce marine carbon emissions, and promote the recycling of waste concrete, the CO_2_-based accelerated carbonation modification of RCA has attracted extensive attention from many scholars worldwide [12,13,14,15]. This method exploits the alkali aggregate properties of RCA for the absorption and sequestration of CO_2_, which greatly promotes resource enhancement and energy savings. The standard carbonation method for RCA based on an environmental temperature of 20 ± 2 °C, a relative humidity of 70 ± 5%, and a CO_2_ concentration of 20 ± 3% has been widely used [16].

Previous efforts have shown that CO_2_-based accelerated carbonation modification methods can promote the microstructural densification of RCA to varying degrees and significantly improve the macroscopic properties of carbonated recycled coarse aggregate (CRCA, see in Table A1 of Appendix A), including the crushing value, apparent density, and water absorption, as well as its microstructures, including phase compositions, pore structures, micromorphology, and OITZ properties [17].

The CO_2_ uptake rate increases as the aggregate particle size (APS, see in Table A1 of Appendix A) of RCA decreases [18,19]. Zhan et al. [20] have reported that the carbonation rate of RCA with an APS of 5–10 mm was much greater than that of RCA with an APS of 14–20 mm. An experimental study by Xuan et al. [21] revealed that the CO_2_ absorption rate of recycled fine aggregates with an APS of less than 5 mm was approximately 2.15%, while that of RCA with an APS ranging from 5 to 10 mm was 0.81%. As the APS of RCA decreased, the specific surface area increased, the adhesion of old attached mortar increased, and the thickness of attachment decreased. As such, the carbonation rate of RCA with small particle sizes increased.

At the same time, the initial moisture condition (IMC, see in Table A1 of Appendix A) of RCA also plays a dominant role in its carbonation efficiency. Theoretically, the optimal initial moisture conditions for the accelerated carbonation of RCA can be determined, and either too high or too low of an IMC has a negative impact on the carbonation efficiency of RCA. Pan et al. [22] have shown that the water absorption of CRCA decreases when the IMC of RCA increases from 2.5% to 5.0%, while the water absorption of CRCA increases when the IMC of RCA reaches 7.5%. Pan et al. [22] further suggested that the best initial moisture condition for accelerated carbonation of RCA is 5.0%, while Zhan et al. [23] suggested that the best initial moisture condition is in the range of 4.0% to 6.5%.

The CO_2_-based accelerated carbonation of RCA can also lead to improvements in the workability, mechanical properties, and durability of RCAC. Tam et al. [24] have reported that the flowability of CRCA mixtures was greater than that of NCA mixtures at an equal water-to-cement ratio and without the addition of additional water, and the maximum increase in the caving degree of the former compared with that of the latter was 57.1% when the replacement rate of RCA was 100%. Similar conclusions were also obtained by Zhang et al. [25]. Moreover, Kou [26] found that the 28 d and 90 d tensile strengths of carbonated recycled aggregate concrete (CRAC, see in Table A1 of Appendix A) increased by approximately 6% and 12%, the flexural strength increased by approximately 28.7%, and the modulus of elasticity increased by 11% to 13.2%, compared with those of ordinary RAC. The analysis revealed that the accelerated carbonation of RCA improved the tensile strength, flexural strength, and modulus of elasticity of the recycled concrete. Xuan [27] has reported that, when the replacement rate of RCA was 100%, the permeability coefficient of CRAC decreased by 43.6% compared with that of ordinary RAC, while the analysis revealed that the carbonation treatment eliminated pore sizes larger than 200 mm and reduced the number of mesopores with sizes from 50 to 200 mm, such that the porosity of RCA decreased. Kou et al. [26] reported that the chloride diffusion coefficient of CRAC decreased by 41% to 46% compared to that of RAC, and similar results were obtained by Xuan et al. [27].

To date, scholars worldwide studying the use of the CO_2_-accelerated carbonation method to enhance the properties of RCA and its concrete have obtained certain research results; however, under different IMCs and APSs of RCA, the degree of improvement of its properties through CO_2_-accelerated carbonation is still unclear. The effect of the best initial moisture condition of RCA on the recycled concrete properties still needs to be explored in depth. For this reason, we carried out experimental research through the preparation of RCA samples with different IMCs and APSs in a standard carbonation environment (by setting an environmental temperature of 20 ± 2 °C, a relative humidity of 70 ± 5%, and a CO_2_ concentration of 20 ± 3%). It is worth noting that there are many potential sources of CO_2_ that can be used for the carbonation of cement substrates and concrete waste, including flue gas or landfill gas [28]. In order to ensure the accuracy of the test results, the CO_2_ used in the test was derived from commercial liquid CO_2_. Through CO_2_-accelerated carbonation, the change rules of RCA properties versus the IMCs and APSs were revealed. The carbonation modification degree of RCA with different IMCs and APSs was investigated, and the best initial moisture conditions for the RCAs corresponding with the optimal accelerated carbonation modification effects were determined. Through the preparation of NAC, RAC, and CRAC specimens with the same mixture ratio and the optimal initial moisture conditions (with the replacement rate of RCA in the latter two samples being 100%), the slumps, compressive strength, and RCM chloride ion diffusion coefficient of different types of concrete samples were measured to investigate the effect of CRCA on the workability, mechanical properties, and durability of the resulting concrete.

The highlights and innovations of this paper include three aspects: 

First, the CO_2_-accelerated carbonation of RCA with different IMCs and APSs revealed changes in properties such as apparent density, water absorption rate, and carbonation rate.

Second, the degree of the accelerated carbonation modification of RCA with different IMCs and APSs was investigated, and the optimal initial moisture condition for the most effective accelerated carbonation modification of RCAs was found to be the completely drying state.

Third, the degree of influence of CRCA on the workability, mechanical properties, and durability of RAC was quantified by performing property tests on NAC, RAC, and CRAC specimens under the optimal initial moisture conditions.

The research results presented in this paper not only serve to improve the properties of RCA and its RCAC as well as reduce the alkalinity of RCA materials and associated negative impacts on the ecological environment, but also allow the alkali aggregation properties of RCA to absorb and solidify carbon dioxide from different sources to be exploited, further promoting resource enhancement, energy savings, and emission reductions, thus having many advantages. 

## 2. Experiment

### 2.1. RCA Production

The RCA samples used in the experiments detailed in this paper were obtained by crushing and sieving concrete with an original strength grade of C40. The mixing ratios of C40 concrete are listed in Table 1. The raw material of the C40 concrete was PC.42.5R composite silicate cement (Chongqing Huaxin Yanjing Cement Co., Ltd., Chongqing, China) produced in the same batch. Continuously graded crushed stone with a nominal particle size of 5–20 mm was used as coarse aggregate, natural freshwater river sand with a nominal particle size of less than 5 mm was used as fine aggregate, and tap water with a density of 1000 kg/m^3^ was used as mixing water. The test result of the concrete’s compressive strength was 41.6 MPa, determined using three parallel specimens, in accordance with its strength grade (i.e., C40).

**Figure 1:**
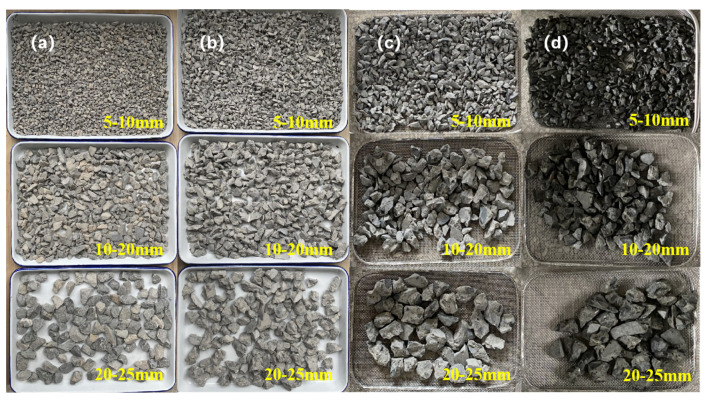
Preparation of coarse aggregate samples with diameters of 5–10 mm, 10–20 mm, and 20–25 mm: (**a**) NCA; (**b**) RCA that was untreated state; (**c**) RCA that was completely drying state; (**d**) RCA that was completely wetting state.

The C40 concrete used in this paper met the ASTM specification “Standard Test Method for Organic Impurities in Fine Aggregate for Concrete (ASTM C40/C40M-20)” [29]. The RCA used in the test was crushed using a jaw crusher, then sieved with an impact standard vibrating sieve machine to obtain RCA samples with APSs of 5–10 mm, 10–20 mm, and 20–25 mm. At the same time, sufficient NCA with the same APS was sieved in a similar manner, and the RCA and NCA samples are shown in Figure 1.

As the accelerated carbonation reaction is carried out in a water environment, the IMC of RCA will affect its carbonation modification effect. Therefore, this study tested this process in combination with the actual situation to consider three kinds of IMCs: completely drying state, untreated state, and completely wetting state. In order to obtain the RCA samples with the above three IMCs, we carried out the following pre-treatment processes, respectively:

To obtain RCA samples in a completely drying state, they were poured into a shallow tray and placed in a blast-dried RCA chamber at 105 ± 5 °C until a constant weight was reached, after which they were removed and cooled to room temperature before being prepared for use.

To obtain RCA samples in their untreated state, they were removed after crushing and sieving without further processing. However, to prevent the atmospheric environment from affecting the samples during the stockpiling process, attention needs to be given to the stockpiling environment.

To obtain RCA samples in a completely wetting state, they were immersed in water for 24 h for full saturation treatment, after which they were removed and a wet towel was used to dry the surface of the specimen to a saturated dry state for use.

### 2.2. Curing Treatment of RCA by Using the CO_2_-Accelerated Carbonation Method

In this study, RCA samples with different APSs and IMCs were tested, and the standard indoor accelerated carbonation test method (with an environmental temperature of 20 ± 2 °C, a relative humidity of 70 ± 5%, and a CO_2_ concentration of 20 ± 3%) was used [16,30,31,32,33] to study the effects of the IMC and APS on the physical properties of CRCA. The actual working conditions are presented in Table 2. In the process of accelerating the carbonation of RCA samples, a standard carbonation chamber was used, where the design model was a TH-W type produced by one-measurement instrument equipment (Hebei) Co., Ltd., Hebei Province, China. The supply of CO_2_ gas came from commercial liquid CO_2_ in a gas storage cylinder, and the preset concentration of CO_2_ gas in the test was supplied to the TH-W type carbonation chamber. The gaseous CO_2_ in the accelerated carbonation environment was maintained at 20 ± 3%.

The specific procedure used for the accelerated carbonation experiments was carried out as described by Wu et al. [30], Yang et al. [31], Ju et al. [32], and Qin et al. [33]. The experimental procedures for RCA samples under accelerated carbonation treatment are listed as follows:

In the first step, the mass of each RCA sample was recorded before carbonation (all with 500 g as the initial mass value). Three parallel samples for each condition were taken and placed in carbonation containers, after which they were simultaneously placed in a concrete carbonation chamber in a standard environment for accelerated carbonation tests.

In the second step, the carbonation chamber was turned on, and the accelerated carbonation treatment experiment for RCA samples was carried out. During this process, the mass variation of each RCA sample was tested after a certain interval of carbonation time, with these operations being more frequent at the beginning of carbonation until the masses of RCA samples remain essentially the constant, at which point the carbonation should be stopped and the test terminated.

In the third step, after complete carbonation of the RCA samples, the three parallel RCA samples were immediately transferred into a drying oven at 105 °C and dried to a constant weight, and the mass of each sample was weighed to determine the moisture content and carbonation ratio of the carbonated recycled coarse aggregate (CRCA). The three parallel samples were subsequently thoroughly mixed and re-divided into three parallel samples to measure the apparent density and water absorption of the CRCA.

### 2.3. Fresh Concrete Cast

Different types of concrete specimens, including the NCAC, RCAC, and carbonated recycled coarse aggregate concrete (CRCAC, see in Table A1 of Appendix A), were prepared according to the mix ratios listed in Table 3, with reference to the “Standard Practice for Making and Curing Concrete Test Specimens in the Field (ASTM C31/C31M-24b)” [34]. The raw materials used for the cement, fine aggregate, water, NCA, and RCA were the same as those in Section 2.1. The RCA samples with different APSs under the optimal initial water content were selected as raw materials, and the CRCA samples obtained after CO_2_-accelerated carbonation were used as coarse aggregate for the preparation of CRCAC.

Initial curing of the concrete specimens was carried out in a standard curing chamber, in which the curing environment temperature was set to 20 ± 5 °C, the relative humidity was set to more than 90%, and the curing time was 1 d. After initial curing was completed, the concrete specimens were demolded using a special demolding pump. Subsequently, the demolded concrete specimens were immediately transferred to a saturated Ca(OH)_2_ solution for 28 d to reach the expected strength grade. After the end of maintenance, the concrete specimens were removed from the Ca(OH)_2_ solution and rinsed using clean water. Subsequently, all specimens were located in a ventilated place to air-dry for 48 h, in preparation for testing of slumps, compressive strength, and chloride permeability, as described in the following Sections. It is worth noting that the final results for the aforementioned three indicators were acquired as mean values based on those obtained from three parallel concrete specimens.

### 2.4. Method

#### 2.4.1. Properties of Coarse Aggregate

##### Apparent Density

The wide-mouth bottle method was used to test the apparent density of RCA samples before and after accelerated carbonation, referring to the specification “Standard Test Method for Bulk Density (“Unit Weigh”) and Voids in Aggregate (ASTM C29/C29M-23)” [35]. The expression for the apparent density of the coarse aggregate is expressed as:

(1) $$\rho_{a}=\frac{m_{1}\cdot \rho_{w}}{m_{1}+m_{2}-m_{3}}$$

where *m*_1_ is the mass of the coarse aggregate samples after completion of drying (g); *m*_2_ is the total mass including the water, the wide-mouth bottle, and the glass piece (g); *m*_3_ is the total mass including the coarse aggregate sample, the water, the wide-mouth bottle, and the glass piece (g); and *ρ_w_* is the density of water (*ρ_w_* = 1000 kg/m^3^).

To quantitatively assess the degree of improvement in the apparent density of RCA using accelerated CO_2_ carbonation, the apparent density difference was defined as follows:

(2) $$\Delta \rho =\rho_{aCRCA}-\rho_{aRCA}$$

Furthermore, the apparent density increase rate Δ*ρ_a_* is:

(3) $$\Delta \rho_{a}=\frac{\Delta \rho }{\rho_{aRCA}}\times 100\%$$

where *ρ_aCRCA_* is the apparent density of *CRCA* (kg/m^3^) and *ρ_aRCA_* is the apparent density of *RCA* (kg/m^3^).

##### Water Absorption

After complete water saturation, the aggregates were dried using a blast drying oven according to the specific test procedure described in the specification “Standard Test Method for Measurement of Rate of Absorption of Water by Hydraulic-Cement Concretes (ASTM C1585-20)” [36]. The formula for water absorption of coarse aggregates is as follows:

(4) $$W_{a}=\frac{m_{4}-m_{1}}{m_{1}}\times 100\%$$

where *m*_4_ is the mass of coarse aggregate with saturated surface dry (g); and *m*_1_ is the same as in Equation (1).

To quantitatively assess the extent to which the carbonation treatment reduces the water absorption of *RCA*, the water absorption difference is defined as follows:

(5) $$\Delta W=W_{aCRCA}-W_{aRCA}$$

Furthermore, the rate of reduction in water absorption Δ*W_a_* is:

(6) $$\Delta W_{a}=\frac{\Delta W}{W_{aRCA}}\times 100\%$$

where *W_aCRCA_* is the water absorption rate of *CRCA* (%) and *W_aRCA_* is the water absorption rate of *RCA* (%).

##### Moisture Content

Moisture content is the physical property of the aggregate that is of the greatest concern in this study, and is also an important index for evaluating how water is transported during the carbonation reaction. Before accelerated carbonation, RCA samples were designed with different water contents and, so, their IMCs needed to be tested. The specific steps are detailed in the specification “Standard Test Method for Measurement of Rate of Absorption of Water by Hydraulic-Cement Concretes (ASTM C1585-20)” [36]. The moisture content in RCA is calculated as follows:

(7) $$\omega =\frac{m_{0}-m_{1}}{m_{1}}\times 100\%$$

where *m*_0_ is the mass of the coarse aggregate samples before drying (g), and *m*_1_ is the same as in Equations (1) and (4). In the conducted experiment, the water content for the RCA was *ω* = 0. 

##### Mass Variation and Actual Mass Variation

The mass variation of RCA characterizes the amount of mass change before and after carbonation of RCA, which still contains a large amount of moisture and needs to be further dried to obtain the actual mass variation; that is, the actual mass variation Δ*m_a_*.

By weighing the masses of RCA samples before and after accelerated carbonation, the mass variation (∆*m*) of RCA after accelerated carbonation can be determined, and the equation for the mass variation of RCA is as follows:

(8) $$\Delta m=m_{c}-m_{0}\cdot \left(1-\omega_{0}\right)$$

where *m_c_* is the mass of RCA after complete carbonization (g); *m*_0_ is the same as in Equation (7); and *ω*_0_ is the initial moisture content of RCA (%). As *ω*_0_ = 0 in our experiment, Equation (8) can be simplified to ∆*m* = *m*_c_ − *m*_0_.

Through further weighing the completely dry mass for CRCA (i.e., *m_cd_*), the actual mass increase in the RCA after accelerated carbonation (i.e., ∆*m_a_*) can be acquired. The formula for ∆*m_a_* is as follows:

(9) $$\Delta m_{a}=m_{cd}-m_{0}\cdot \left(1-\omega_{0}\right)$$

where *m_c__d_* is the actual mass variation of RCA after accelerated carbonation (g); *m*_0_ is the same as in Equations (7) and (8); and *ω*_0_ is the same as in Equation (7).

##### Carbonation Ratio

The carbonation ratio is the degree of carbonation of RCA, which has been previously studied by many scholars. The carbonation ratio for RCA can be calculated using the following equation:

(10) $$C_{ratio}=\Delta m_{a}/\Delta m_{T}\times 100\%$$

where ∆*m_a_* is the actual mass variation of RCA after completion of carbonation (g), as elaborated in Equation (5), and ∆*m_T_* is the actual mass variation of RCA after completion of carbonation (g), which has been confirmed in detail in a previous study [37].

#### 2.4.2. Properties of the Concrete

##### Slumps

Concrete mix compatibility properties are usually assessed using a slump test. The slump test procedure for the NAC, RAC, and CRAC specimens in this experiment was implemented according to the specification ASTM C143/C143M-20 [38].

##### Compressive Strength

Compressive strength is one of the most basic indicators for assessing the mechanical properties of concrete, and in this study, the test procedure for the 28 d cubic compressive strength of NAC, RAC, and CRAC was implemented according to the specification ASTM C39/C39M-24 [39].

##### Chloride Permeability

**Figure 2:**
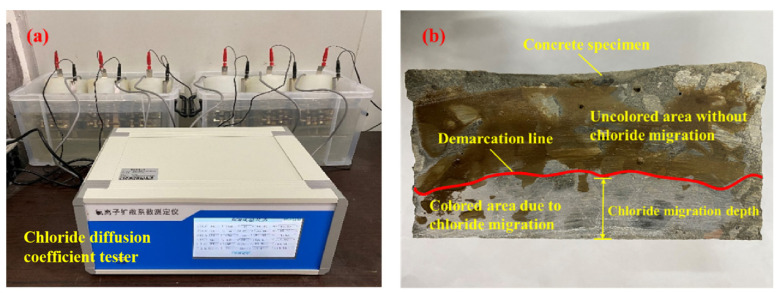
RCM electromigration test: (**a**) RCM chloride diffusion coefficient test; (**b**) chloride electromigration depth determination.

Concrete’s resistance to chloride permeability is determined by measuring the unsteady-state migration coefficient of chloride ions in concrete using the unsteady-state rapid chloride ion electromigration assay (RCM method). In this study, the RCM chloride ion diffusion coefficient was used as an index to evaluate the durability of various types of concrete, where the test method refers to the specification ASTM C1202-22e1 [40]. The test process is depicted in Figure 2.

The RCM chloride diffusion coefficient of concrete is calculated using the following equation:

(11) $$D_{RCM}=\frac{0.0239\times \left(273+T\right)L}{\left(U-2\right)t}\left(X_{d}-0.0238\sqrt{\frac{\left(273+T\right)LX_{d}}{U-2}}\right)$$

where *D_RCM_* is the unsteady-state chloride ion migration coefficient of concrete, which is accurate to 0.1 × 10^−12^ m^2^/s; *T* is the average value of the initial and final temperatures of the anode solution (°C); *L* is the height of the cylindrical concrete specimen, which is accurate to 0.1 mm; *U* is the absolute value of the applied voltage in the test (V); *t* is the time of energization in the test (h); and *X_d_* is the average chloride ion electromigration depth, which is accurate to 0.1 mm.

## 3. Results, Analysis, and Discussions

### 3.1. Effect of APSs and IMCs on the Properties of CRCA

#### 3.1.1. Apparent Density

**Figure 3:**
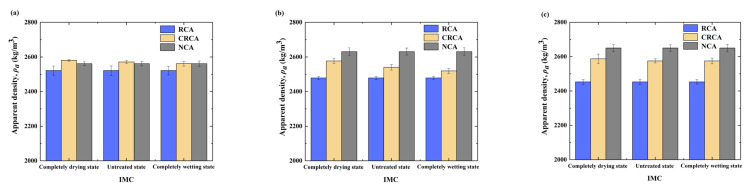
Measured apparent densities of NCA, RCA and CRCA with different IMCs and APSs: (**a**) 5–10 mm, (**b**) 10–20 mm, (**c**) 20–25 mm.

**Figure 4:**
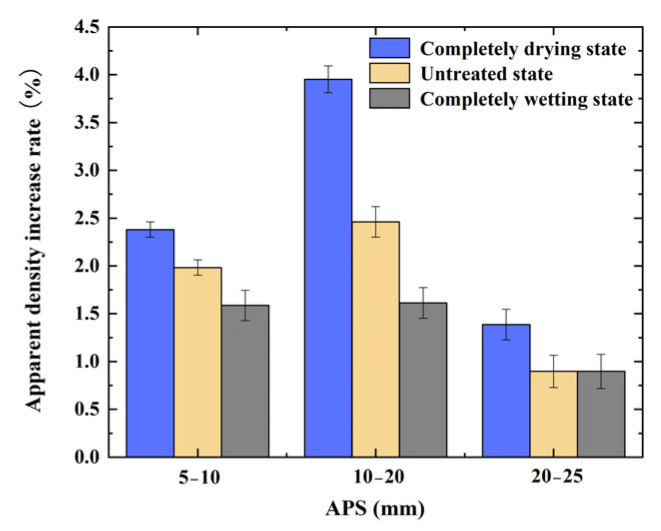
Increase in apparent density before and after the accelerated carbonation of RCA samples with different IMCs and APSs.

The apparent densities of the NCA, RCA, and CRCA samples with different APSs are presented in Figure 3 and Figure 4.

Figure 3 shows that the apparent density of NCA increased in a near-linear manner with increasing APS. Nevertheless, the apparent density of RCA exhibited completely opposite trends to that of NCA; specifically, the apparent density of RCA decreased nearly linearly as the APS increased. Additionally, the apparent densities of CRCA also showed a decreasing trend with increasing APS; however, the degree of change for CRCA demonstrated differences from those for NCA and RCA. The apparent density of the 10–20 mm CRCA was reduced by only approximately 0.13%, compared with that of the 5–10 mm CRCA, whereas the apparent density of the 20–25 mm CRCA was decreased by approximately 3.51% when compared with that of the 10–20 mm CRCA, which was the largest reduction among all the situations.

As shown in Figure 3, at the same IMC, the apparent density of NCA was the highest; the apparent density of RCA was the lowest for the same APS; and the apparent density of CRCA was between those of NCA and RCA. The apparent density of the RCA samples at the same IMC decreased by approximately 6.02%, 8.54%, and 10.35% when compared with the 5–10 mm, 10–20 mm, and 20–25 mm NCA samples, respectively, further demonstrating that the apparent density of the RCAs was worse than that of the NCA. Moreover, the reduction in the apparent density of RCA compared to that of NCA gradually increased as the APS of the coarse aggregate increased. The apparent density of the CRCA with different APSs was approximately 2.40%, 3.97%, and 1.40% greater than those of the respective RCAs, indicating that the accelerated carbonation experiment based on CO_2_ can effectively improve the apparent density of the RCA and, hence, enhance its properties. The maximum apparent density enhancement was observed for the CRCA with an APS of 10–20 mm, indicating the optimal accelerated carbonation modification effect.

After analysis, the reason for the above phenomenon is that the RCA samples with the APS of 5–10 mm are less attached to the old mortar, resulting in less hydration that can participate in the carbonation reaction; as such, the RCA carbonation reaction is insufficient, such that its performance improvement under standard carbonation conditions is worse than that of the 10–20 mm samples. In addition, although the RCA samples with the APS of 20–25 mm were attached to a thicker layer of old mortar and had more hydration that could participate in the carbonation reaction, their larger particle size resulted in a smaller specific surface area than that of the 10–20 mm samples [5]. As such, the accelerated carbonation modification effect of the RCA with the APS of 20–25 mm was inferior to that of the 10–20 mm samples. Similar results have also been obtained by Wu et al. [30].

As shown in Figure 4, the largest increase in apparent density for the RCAs with the different IMCs and the same APS occurred for the RCA with an APS of 10–20 mm and a completely drying state, indicating that, in the standard carbonation environment, minimizing the IMC of the RCA can improve its carbonation modification effect. Similar results have been reported by Ju et al. [32]. In summary, for RCA samples with an APS of 10–20 mm, the increase in the apparent density is the greatest, leading to the best carbonation effect; however, the IMC of the RCA is a key factor affecting the rate of carbonation.

#### 3.1.2. Water Absorption

**Figure 5:**
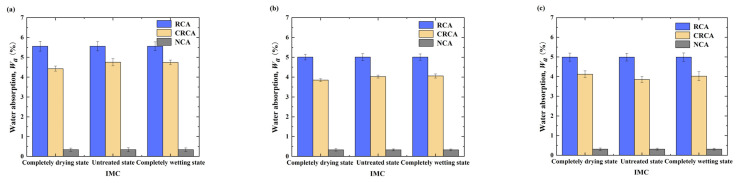
Measured water absorption of NCA, RCA and CRCA at different IMCs and APSs: (**a**) 5–10 mm, (**b**) 10–20 mm, (**c**) 20–25 mm.

**Figure 6:**
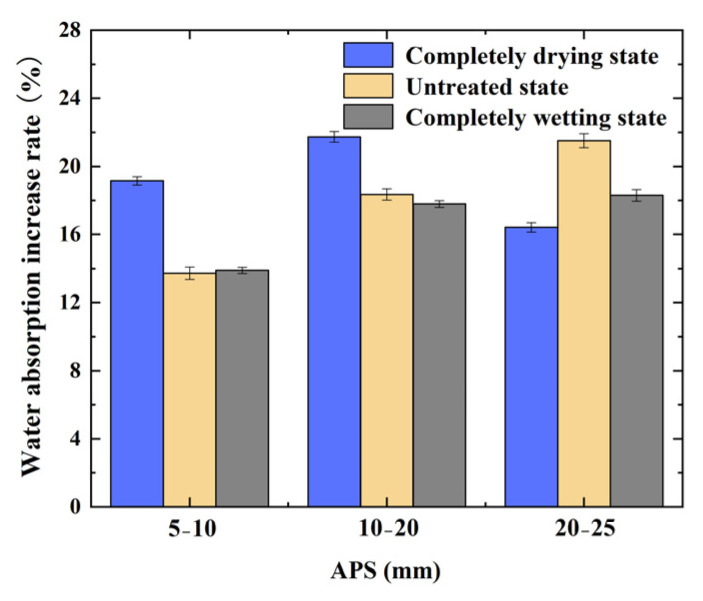
Reduction rate of water absorption before and after accelerated carbonation of RCA with different initial IMCs and APSs.

Next, the water absorption results for the NCA, RCA, and CRCA with different APSs were determined, and are shown in Figure 5 and Figure 6.

As shown in Figure 5, the water absorption values for NCAs were the lowest, while those for RCA were highest at the same APS, and the measurements for CRCA were between those of NCA and RCA. The tested water absorption values of the RCA samples were 16.18, 15.2, and 15.9 times greater than those of the NCA samples with sizes of 5–10 mm, 10–20 mm, and 20–25 mm, respectively, indicating that the water absorption values of the RCA samples were much greater than those of the NCA samples.

The water absorption of CRCA with different APSs was reduced by approximately 19.16%, 21.8%, and 16.3%, respectively, compared to the associated RCA, further demonstrating that the accelerated carbonation method based on CO_2_ can effectively decrease the water absorption of RCA and thus improve its properties. The maximum reduction in CRCA water absorption was also observed at an APS of 10–20 mm, further indicating the optimal accelerated carbonation modification effect.

As shown in Figure 6, by analyzing the rule governing the change in the water absorption of the CRCA samples with different IMCs, the IMC of RCA is the key factor affecting the change in the water absorption rate, which determines the overall quality development trend of the CRCA. The optimal IMC was the completely drying state, and the modification effect of the RCA with an APS of 10–20 mm was the greatest.

According to the analysis, this is because there is still a large amount of water when the RCA samples are in the untreated or completely wetting states, hindering the diffusion of CO_2_ into the samples under a standard carbonation environment, resulting in improved water absorption values for the untreated and completely wetting state RCA samples being lower than those of the dried RCA samples. Ju et al. [32] also obtained a similar change rule through experimental research.

#### 3.1.3. Moisture Content

**Figure 7:**
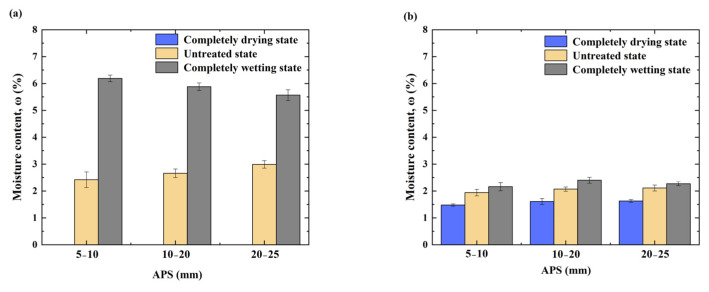
Measured moisture content before and after accelerated carbonation of RCA at different IMCs and APSs: (**a**) RCA, (**b**) CRCA.

As shown in Figure 7, the moisture content of the RCA increased gradually with increasing APS for the completely drying and untreated state conditions; for the completely wetting state condition, the moisture content of the RCA decreased gradually with increasing APS.

Due to the large humidity gradient between the RCAs subjected to the completely drying state and the test environment, CO_2_ and water can more easily penetrate the internal RCA and, after the carbonation treatment and absorption of water, the moisture content is significantly increased; moreover, the smaller the APS of the RCA in the untreated state and completely wetting state, the fewer hydration products the RCA contains and the lower its ability to absorb water in the carbonation process. The moisture content of the RCA after carbonation was reduced to different degrees. Compared with that of the untreated state RCA, the moisture content of the RCA in the completely wetting state was reduced to the greatest extent. Figure 7 shows that, at the same APS, the lower the IMC of the RCA, the more water it can absorb from the environment and the greater the degree of increase in its moisture content after carbonation.

According to the analysis, as the pore humidity of the completely drying state RCA is much lower than that of the carbonized environment, there is an obvious humidity gradient difference between the inside and the outside, and the water in the environment can easily penetrate into the RCA; thus, the moisture content of the RCA after carbonation is greatly increased. However, as the pore humidity of saturated RCA is much higher than that of the carbonized environment, the internal and external humidity gradient of RCA is reversed, compared with that of dried RCA, resulting in a decreasing trend in moisture content after carbonation. In summary, the change in the moisture content of the RCA samples depends greatly on the relative humidity in the carbonation environment and the moisture content of the RCA; furthermore, the interaction between these two factors affects the properties of the resulting CRCA.

#### 3.1.4. Mass Variation and Actual Mass Variation

**Figure 8:**
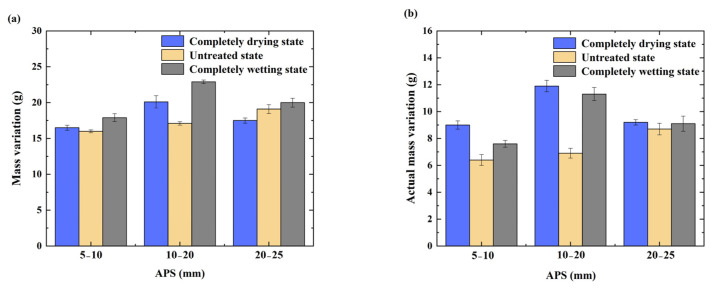
Mass variation and actual mass variation of CRCA with different IMCs and APSs: (**a**) mass variation, (**b**) actual mass variation.

As shown in Figure 8, the mass variation and actual mass variation of the RCA in the completely drying and completely wetting states after accelerated carbonation increased and then decreased with increasing APS, while the mass variation and actual mass variation of the RCA in the untreated state after carbonation increased with increasing APS. In addition, the mass variation and actual mass variation of the RCAs with different IMCs (i.e., completely drying state or completely wetting state) and the same APS were greater than those of the RCAs in the untreated state, as the humidity in the pores of the RCAs in the completely drying state was much lower than that in the carbonation environment. The difference between the internal and external humidity was obvious, and the water in the environment carried CO_2_, which easily penetrated into the interior of the RCA, which was beneficial for the carbonation reaction. Meanwhile, the humidity in the pores of the water-saturated RCAs was much greater than that in the carbonation environment, resulting in a smaller degree of carbonation; however, the rate of CO_2_ penetration was still greater than that in the untreated state sample, which caused the water-saturated RCAs to have greater mass variation and actual mass variation than the samples in the untreated state. The mass variation and actual mass variation of the CRCA in the completely drying, untreated, and completely wetting states were greatest with an APS of 10–20 mm.

#### 3.1.5. Carbonation Ratio

**Figure 9:**
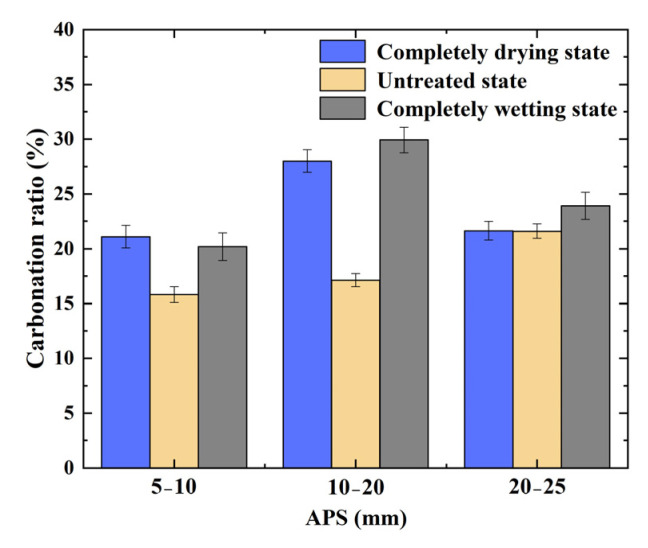
Carbonation ratio of RCA with different APSs and different IMCs.

Figure 9 shows that, when the IMC was in the completely drying or completely wetting state, the carbonation ratio of the RCAs first increased and then decreased with increasing APS, and the carbonation ratio of the RCAs was the highest when the APS was 10–20 mm, while that for the untreated state samples increased with increasing APS. The relative reasons for the aforementioned phenomena are consistent with the previous analysis of RCA apparent density modification.

When the APS was constant, the carbonation ratios of the RCAs in the completely drying and completely wetting states were greater, where the mean values of the carbonation ratio reached 23.59% and 24.67%, respectively; notably, these were very close to each other in value. The lowest value was obtained for the RCA in the untreated state, where the mean value of its carbonation ratio was 18.18%, as exhibited in Table 4. The aforementioned variation trends are similar to those reported by Zhan et al. [20]. In summary, the RCAs with the same APS in the completely drying/wetting state exhibited the best carbonation effect.

Through analysis, this is because the pore humidity in the completely drying state (completely wetting state) RCA is much lower than (much higher than) the relative humidity in the carbonation environment, creating an obvious positive (negative) humidity gradient between the carbonation environment and the inside of the RCA samples. In this case, the solute in the carbonation reaction is accelerated; that is, if the amount of water (environmental relative humidity or RCA pore water) is sufficient, CO_2_ is more likely to penetrate into the interior of RCA, and the carbonation reaction occurs, resulting in a higher carbonation degree and a higher carbonation rate of completely drying or completely wetting RCA. The above conclusions are consistent with those of Wu et al. [30].

### 3.2. Best Conditions for RCAs Based on CO_2_ Accelerated Curing Treatment

After comprehensively analyzing the variation trends of the apparent density, water absorption, moisture content, mass variation, and carbonation ratio of the RCAs before and after carbonation, as detailed in Section 3.1, the IMCs corresponding to RCAs with different APSs leading to the best carbonation and modification effect are summarized in Table 5.

**Figure 10:**
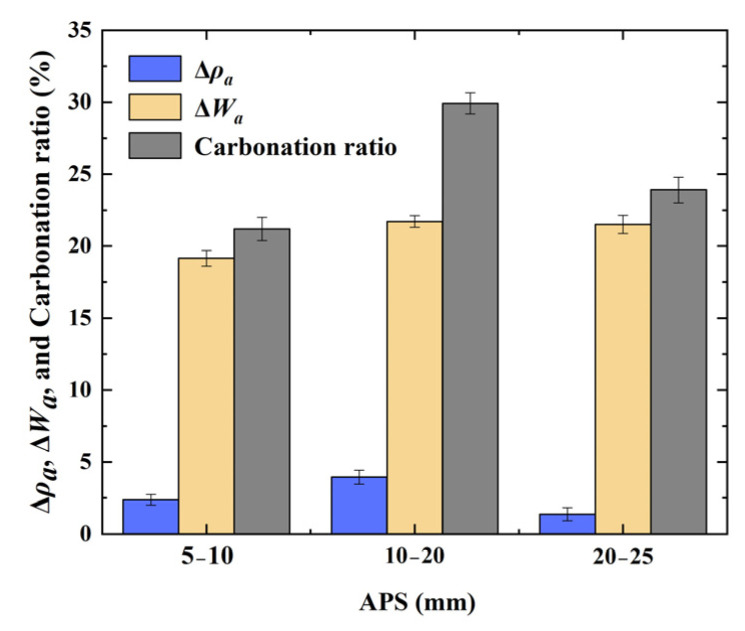
Property indices before and after carbonation for the RCA with different APSs corresponding to the best initial moisture condition.

Table 5 shows that the correspondence between the APS and IMC for the best carbonation modification effect of the RCA was 5–10 mm and a completely drying state; 10–20 mm and a completely drying state; and 20–25 mm and a completely drying state. Therefore, the optimal initial moisture condition was a completely drying state for the RCA samples with different APSs. The apparent density increase ratio, water absorption reduction ratio, and carbonation ratio of the RCA samples with different APSs under the completely drying state IMC are plotted in Figure 10, indicating that the improvement in these indices was the greatest for the RCA with an APS of 10–20 mm, compared to 5–10 mm or 20–25 mm.

In summary, the IMC and APS of the RCAs with the best carbonation modification effect are completely drying state and 10–20 mm, respectively.

### 3.3. Improvements in the Properties of CRCAC

CRCAs with different APSs under the optimal carbonation modification conditions (completely drying state) described in Section 3.2 were prepared according to Section 2.3 to obtain sufficient CRCAC. Then, the slump, compressive strength, and RCM chloride diffusion coefficient of the NCAC, RCAC, and CRCAC samples were obtained. A comparative analysis of the effect of the CRCA on the properties of the CRCAC was conducted as follows.

#### 3.3.1. Slumps

**Figure 11:**
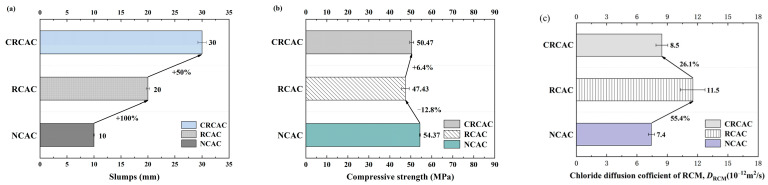
The test results for the different concrete mixtures: (**a**) slumps, (**b**) compressive strength, (**c**) chloride diffusion coefficient of RCM.

Concrete mixtures are usually evaluated through slump tests, and the results from the NAC, RAC, and CRCAC mixtures are shown in Figure 11a. As shown in Figure 11a, at the same water–cement ratio, the NAC mixture had the smallest slump, which was 10 mm, and the slump values of the RAC and CRCAC were greater than those of the NAC. The slump of the RAC was 100% greater than that of the NAC (which was 20 mm), and that of the CRCAC was 50% greater than that of the RAC (which is 30 mm).

The workability of both recycled concrete mixtures was better than that of NAC, as the pre-saturated treatment of RCA and CRCA prior to mixing resulted in more free water remaining in the mortar during the mixing process, which increased the fluidity of the aggregates. CRCAC had better workability than RAC as the water absorption of RCA is significantly reduced by carbonation due to denser pores and decreased crack channels; furthermore, the precipitated free water also remains in the mortar, which increases the slump value of the recycled aggregate concrete. The aforementioned research results were in line with those of Zhang et al. [25] and Wu et al. [41].

#### 3.3.2. Compressive Strength

Figure 11b shows the measured 28 d cubic compressive strengths of the NAC, RAC, and CRCAC specimens. As shown in the figure, at the same water–cement ratio, the compressive strengths of the RAC and CRCAC were lower than that of the NAC, in which the relative difference between the compressive strength of the RAC and that of the NAC was 12.8%, which was due to the crushing process performed for RCA preparation, which damaged the mechanical properties of the aggregates themselves, resulting in the compressive strength of the RAC being less than that of the concrete prepared from the NCA.

In addition, the weak phase of RAC, i.e., the old mortar and OITZ attached to the RCA, also significantly reduced the compressive strength of RAC. Compared with the RAC, the compressive strength of the CRCAC was increased by 6.4%, which indicates that CRCA can improve the mechanical properties of recycled concrete, to a certain extent, as the carbonized products (e.g., CaCO_3_ and silica gel) compact the pores and cracks of the old mortar on the surface of the RCA, resulting in a significant improvement in the pore structure. At the same time, it can be seen from Section 3.1 of this paper that the apparent density of RCA increased significantly after CO_2_-accelerated carbonation, which was another important reason for the increase in compressive strength and mechanical properties of CRCAC specimens. Similar conclusions were mentioned in the study of Wu et al. [41].

#### 3.3.3. Chloride Permeability

The results of the RCM chloride diffusion coefficient tests for NAC, RAC, and CRCAC specimens are shown in Figure 11c. Figure 11c shows that, at the same water–cement ratio, the D_RCM_ of NAC was the lowest (at 7.4 × 10^−12^ m^2^/s), while the D_RCM_ of RAC was the highest at (1.15 × 10^−11^ m^2^/s), comprising an increase of 55.4% in comparison with that of NAC.

This was due to the water absorption of RCA being 15–18 times that of NCA, such that its internal penetrating holes provide more channels for chloride ion penetration, thereby increasing the chloride ion electromigration depth X_d_ of RCA, making RAC poor in chloride ion penetration resistance and durability in comparison to NAC. Compared with RAC, the D_RCM_ of CRCAC was reduced by 26.1%; that is, CRCA reduces the chloride ion permeability of recycled concrete. This is due to the carbonized aggregate compacting the pores of RCA and blocking the pore channels for chloride ion transport, thus improving the pore structure of CRCAC and reducing the porosity of the material. The chloride permeability of CRCRA was significantly reduced, and its resistance to chloride ion penetration was improved. Zhang et al. [25], Xuan et al. [27], and Wu et al. [41] have all reported highly similar research conclusions.

### 3.4. Summaries and Discussions

The main reason for the improved performance of CRCA compared with RCA is that the internal pores of RCA are more compact, and the mesostructure is significantly improved after accelerated carbonation with CO_2_. As the internal and microscopic pore densities of RCA samples with different IMCs and APSs after accelerated carbonation differ, the improvement degree of CRCA macro-properties (e.g., apparent density, water absorption, actual mass variation, and carbonation ratio) also differs. However, through comprehensive analysis, we still found that, when the IMC of RCA was in a completely drying state, the modification effect of the prepared CRCA was optimal. On this basis, we took RCA samples with different particle sizes and the completely drying state of the IMC as raw materials and prepared CRCA through accelerated carbonation, which was further used to form CRCAC specimens. According to the test results, the slump, compressive strength, and chloride permeability of CRCAC specimens—which are three important indices reflecting the workability, mechanical properties, and durability of concrete—were improved compared with those of RCAC.

The analysis shows that, when the conditions of cement, river sand, water, and other admixtures are consistent, the main factors affecting the performance of concrete materials depend on the characteristics of the coarse aggregate. For NCAC, as NCA has the highest apparent density, the lowest water absorption, and the best performance in all aspects, NCAC prepared with NCA undoubtedly obtained the best performance indices. For unmodified RCA, due to the old mortar and the large number of pores and microfracture channels in the OITZ, the prepared RCAC had high water absorption and low apparent density, and so, the RCAC prepared with RCA had the worst performance characterization in all aspects. The CRCA obtained after accelerated carbonation of the RCA with CO_2_ contained a large number of densification products, such as calcium carbonate and silica gel, through its internal carbonation reaction, which effectively compacts the pores and blocks the micro-crack channels, improves the microstructure of the RCA, reduces the water absorption rate, increases the apparent density, and effectively improves the performance indicators in all aspects. Therefore, although the performance of CRCAC prepared from CRCA still lagged behind that of NCAC, it was significantly improved and enhanced when compared with RCAC.

## 4. Conclusions and Outlooks

Through an experimental study focused on the carbonation modification of RCAs with different APSs under completely drying, untreated, and completely wetting IMCs, the effects of the IMC and APS of RCAs on indices such as the apparent density, water absorption, moisture content, mass variation and actual mass variation, and the carbonation ratio of RCAs before and after carbonation modification were clarified. Through an in-depth analysis of the experimental data, the IMC of the RCA with the optimal carbonation effect was determined to be the completely drying state, and the corresponding CRCA was used to replace 100% of the natural aggregate and was prepared into CRCAC specimens. The performance of the CO_2_-based RCAC was comparatively assessed through measurement of the slump, compressive strength, and RCM chloride diffusion coefficient values of the NCAC, RCAC, and CRCAC specimens. The accelerated carbonation method was used to evaluate the degree of improvement of the RAC in terms of workability, mechanical properties, and durability, and the obtained conclusions are as follows:

First, the degree of carbonation modification of the RCAs increased with decreasing APS. The apparent densities of CRCA with APSs of 5–10 mm, 10–20 mm, and 20–25 mm were 2.40%, 3.97%, and 1.40%, respectively—greater than those of RCAs with the same APS—and the water absorption decreased by approximately 19.16%, 21.8%, and 16.3%, respectively, indicating that the accelerated carbonation method based on gaseous CO_2_ can effectively improve the properties of RCA.

Second, the lower the IMC of the RCA, the more it can capture CO_2_ and absorb moisture from the environment; that is, when the IMC of the RCA was in the completely drying state, the RCA exhibited better modification after accelerated carbonation, and the modification effect was the worst when the IMC was in the untreated state. It is suggested that reducing the IMC of RCA can have positive effects on its carbonation modification in a carbonation environment with high humidity.

Third, the best optimal moisture conditions for the RCAs with different APSs were determined to be the completely drying state. In addition, compared with the RCAs with APSs of 5–10 mm and 20–25 mm, the RCA with an APS of 10–20 mm showed the greatest improvement in all considered indices. Therefore, the IMC and APS of the RCA with optimal carbonation modification effects were determined to be the completely drying state and 10–20 mm, respectively.

Fourth, carbonation of the RCAs improved the compatibility, mechanical properties, and durability of the RAC to a certain extent. The slump and compressive strength values of the CRCAC were 50% and 6.4% greater than those of the RCAC, respectively, while the D_RCM_ of the CRCAC decreased by 26.1% compared to that of the RCAC. This is because the carbonation modification reduces the absorption of water by the RCA and optimizes the pore structure of the RCA, reducing the porosity of the resulting CRCAC and the number of permeation channels for chloride ions, leading to improvements in the workability, mechanical properties, and durability of the RCAC.

In view of the realistic problem of the limited ability to exploit natural resources such as natural sand and stone in the future, the popularization and application of RCA technology using solid waste resources as raw materials in practical engineering is bound to become the prevailing trend. Although the insufficient performance of RCA restricts the popularization and application of this material, the feasibility and effectiveness of using accelerated carbonation methods to improve the performance of RCA and produce CRCAC products were demonstrated in this paper. With the continuous acceleration of urbanization, the amount of demolished and discarded concrete is increasing annually, which provides a solid foundation for the stable supply of RCA as a raw material. At the same time, the large amount of waste gas CO_2_ produced by the cement industry can be stored and reused through relevant technological means, allowing for the large-scale accelerated carbonation of RCA and the preparation of CRCA and its CRCAC as finished products. In this process, the waste CO_2_ and the waste RCA after crushing and screening are placed in a large carbonation silo, and RCA with different particle sizes can be pre-treated in a completely drying state, ensuring the optimal carbonation modification of the RCA. Considering the adequacy of raw material sources, the resource utilization of waste gas CO_2_, and the controllability of carbonation reaction conditions, we agree that the large-scale production of such low-carbon environmental protection CRCAC is not only technically feasible but also in line with the concepts of energy conservation, emission reduction, and sustainable development, which can lay a solid foundation for achieving the global “double carbon goal”.

## Figures and Tables

**Table 1:** Mix proportions of C40 concrete for RCA production.

Strength	w/c	Water (kg/m^3^)	Cement (kg/m^3^)	River Sand (kg/m^3^)	Crush Stone (kg/m^3^)	Sand Rate (%)	Tested Compressive Strength (MPa)
C40	0.5	195	390	617	1198	0.34	41.6
		8.13%	16.25%	25.71%	49.91%		

**Table 2:** Different operation conditions for the RCA test.

Original Concrete Strength (MPa)	Aggregate Particle Size APS (mm)	Initial Moisture Condition IMC	Initial Moisture Content (%)
C40	5~10	Complete drying state	2.00
		Untreated state	5.84
		Complete wetting state	0.00
	10~20	Complete drying state	2.00
		Untreated state	5.84
		Complete wetting state	0.00
	20~25	Complete drying state	2.00
		Untreated state	5.84
		Complete wetting state	0.00

**Table 3:** Standard mix ratio of the newly mixed concrete (kg/m^3^).

Concrete Type	Water-to-Cement Ratio	Water	Cement	Fine Aggregate (Sand)	Coarse Aggregate
NCAC	0.4	195	488	584	1134
RCAC					
CRCAC					

**Table 4:** Mean and standard deviation of the carbonation ratio of the RCAs for each IMC.

Initial Moisture Condition	Mean Value (%)	Standard Deviation
completely drying state	23.59	3.14
untreated state	18.18	2.48
completely wetting state	24.67	4.00

**Table 5:** APS and IMC corresponding to the best carbonation modification effects for RCA.

APS (mm)	Δ*ρ_a_* (%)	Δ*W_a_* (%)	Actual Mass Variation (g)	Carbonation Ratio (%)
5–10	completely drying state	completely drying state	completely drying state	completely drying/wetting state
10–20	completely drying state	completely drying state	completely drying state	completely drying/wetting state
20–25	completely drying state	untreated state	completely drying state	completely drying/wetting state

## Data Availability

Data are contained within the article.

## Appendix A

**Table A1:** Symbol descriptions for this paper’s acronyms.

Acronyms	Full Name	Acronyms	Full Name
RCA	Recycled coarse aggregate	NCA	Natural coarse aggregate
RCAC	Recycled coarse aggregate concrete	CRCA	Carbonated recycle coarse aggregate
APS	Aggregate particle size	IMC	Initial moisture condition
OITZ	Old interfacial transition zone	NCAC	Natural coarse aggregate concrete
CRAC	Carbonated recycle aggregate concrete	CRCAC	Carbonated recycle coarse aggregate concrete
